# Leachate effects of pelagic *Sargassum* spp. on larval swimming behavior of the coral *Acropora palmata*

**DOI:** 10.1038/s41598-020-60864-z

**Published:** 2020-03-03

**Authors:** Francisco Antonio-Martínez, Yann Henaut, Alejandro Vega-Zepeda, Ana I. Cerón-Flores, Rodolfo Raigoza-Figueras, Neidy P. Cetz-Navarro, Julio Espinoza-Avalos

**Affiliations:** 1 ECOSUR, Avenida Centenario km 5.5, Colonia Pacto Obrero Campesino, Chetumal, 77014 Quintana Roo Mexico; 2 Acuario XCARET, Playa del Carmen, 77710 Quintana Roo Mexico; 3 Present Address: Freelance, Chetumal, 77025 Quintana Roo Mexico

**Keywords:** Behavioural ecology, Conservation biology, Behavioural ecology

## Abstract

An emerging disturbance for Caribbean reefs is the massive arrival of pelagic *Sargassum*, which deteriorates water quality due to the production of leachates. The highest arrivals of *Sargassum* took place when broadcasting corals spawned. We experimentally determined the effect of *Sargassum* leachates on swimming behavior of *Acropora palmata* larvae through five treatments (control, stain (simulating 100% leachate color), and 25%, 50% and 100% *Sargassum* leachate concentrations) during 30 min (10 min of videos and 20 min of post-observations). In the videos, larvae with leachates reduced swimming speed, were positively geotactic, the percentage of individuals that swam in a spiral pattern increased, and most behavioral displacements occurred at lower frequencies than larvae without leachates. Moreover, symptomatic spiral behavior was higher in the presence of leachates, suggesting that this behavior may be an effect of pollution. During post-observations, most larvae with leachates were motionless. This is the first time that *Sargassum* leachates have been documented modifying larval swimming behavior, which may reduce larval dispersion and genetic diversity. We suggest that a future evaluation of the effects of leachates at lower concentrations and over longer periods of exposure is needed. The resilience of corals may be compromised if *Sargassum* arrivals become frequent events.

## Introduction

Globally, coral reefs are being degraded by several stressors (e.g., climate change, bleaching, overfishing and emergent diseases^1–5^), especially those in the Caribbean^6^. An emerging stressor for Caribbean reefs is the massive arrival of two pelagic macroalgal species: *Sargassum fluitans* and *S*. *natans*. Pelagic *Sargassum* drifts on the surface of the sea, providing an important habitat for a great diversity of organisms^7,8^. The presence of a high biomass of pelagic *Sargassum* spp. began in 2011 in the central Atlantic Ocean and the Caribbean Sea^9^. However, *Sargassum* influx in the Mexican Caribbean has only occurred since 2014, with the highest peaks reported in September 2015 (~2360 m^3^ algae km^−1^) and May 2018 (8793 m^3^ algae km^−1^)^10^. Unfortunately, the massive arrival of pelagic *Sargassum* is predicted to become a recurring disturbance^9^.

**Figure 1:**
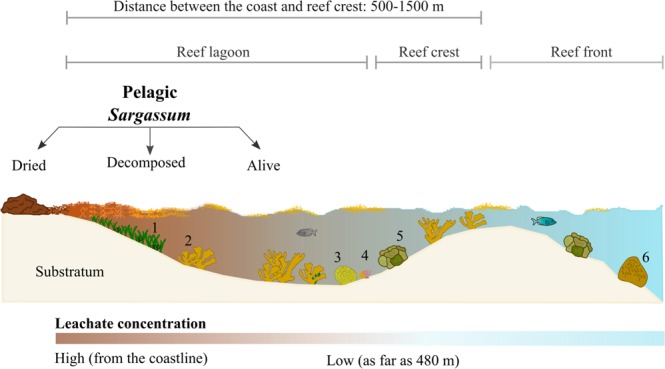
Schematic profile at Punta Venado, Quintana Roo, Mexico. Here, thick caps of pelagic *Sargassum* that accumulate at the coastline, produce leachates when decomposing. Leachate concentration decreases as distance from the coast increases. In addition, *Acropora palmata* was found as far away as 60 m from the coastline. 1 = seagrass meadows mixed with seaweeds. Stony corals are represented by reef-building and opportunistic corals. Reef-building corals: 2 = *A*. *palmata*; 3 = *Pseudodiploria*; 5 = *Orbicella annularis*; and 6 = *O*. *faveolata*. Opportunistic corals: 4 = *Agaricia* and *Porites*. Based on^10,11,58^ and pers. obs. in the field.

Decomposition of the accumulated biomass of pelagic *Sargassum* in the coastline produces leachates that reduce dissolved oxygen (DO) and pH, and increase turbidity and temperature, deteriorating water quality^10,11^. These leachates create conditions similar to eutrophication and may contain heavy metals such as mercury (Hg) and methylmercury (MeHg)^12^, methane^13^, lactic acid^14^, and high concentrations of ammonium and phosphorus^10^. In the Mexican Caribbean, many coral species inhabit lagoon reefs (i.e., near to the coastline) where leachates of pelagic *Sargassum* have been observed and dispersed from the coastline toward the reef crest. In this context, some coral species became stressed and presented partial or total mortality due to the presence and decomposition of *Sargassum*^11^ (Fig. 1).

Brooding and broadcasting stony coral species display different reproductive strategies to colonize reefs. Brooding corals produce few zooxanthellae larvae that settle within hours close to their parents. In contrast, broadcasting corals release a lot of gametes (some species may produce billions) with external fertilization. After embryogenesis, larvae lacking zooxanthellae may swim for days or weeks^15^. Cilia allow coral larvae to disperse several km away from their parents; for instance, larvae of *Acropora* spp. may travel up to 720 km^16^. Long-range dispersal of coral larvae may function as a way to reduce inbreeding^17^, to colonize new environments with lower competition^15^, to recolonize other reefs^16^ or to escape from unfavorable environments^17^. In addition to larval swimming behavior (e.g., vertical and horizontal displacements), external factors (e.g., current direction and turbidity) may influence larval dispersion^15,18^. Larval swimming behavior has been used to assess the response of early coral stages to different environmental conditions or stressors such as oil^19^, oil dispersants^20^, allelopathic algae compounds^21,22^, nutrient enrichment, temperature increase^23^, and the presence of macroalgae^24^ and cyanobacteria^25^. In these studies, larval changed in terms of swimming patterns (linear, sinuous, circular, immersion, spiral, swinging, up, down, with stops, in reverse and random), swimming speed (mm/s), evaluations or tests (‘tests’) of the substrate, displacement along the bottom and settlement capacity. Trajectometry analysis in animals (evaluating how individual animals move through space and time in two dimensions) has also been used to answer questions related to foraging, navigation, dispersion ecology, migration, and mimicry of animal behavior^26^.

*Acropora palmata* is a primary element for coral reef formation in shallow areas of the Caribbean Sea^27^. This hermaphrodite species synchronously release bundles with both gametes in an annual spawn that may occur from July to September^28^. This coral species is classified as “Critically Endangered” by the International Union for the Conservation of Nature (IUCN)^29^ and “Subject to Special Protection” by NOM-059-SEMARNAT-2010 in Mexico^30^. Emergent diseases^31–33^, bleaching^2^, and hurricanes^31,34^ have decimated its populations with low rates of recovery^35^. Moreover, population increases are predominantly a result of asexual^36^ rather than sexual reproduction because early life stages (e.g., coral larvae) are vulnerable^22,23,37,38^. Due to the massive inputs of pelagic *Sargassum* in the Mexican Caribbean reefs in recent years^10,11^, *Sargassum* leachates may be another source of stress that may reduce the income of *A*. *palmata* sexual recruits.

Here, we exposed competent larvae of *A*. *palmata* to five treatments (control, stain (caramel artificial food color simulating 100% leachate color), and 25%, 50%, and 100% *Sargassum* leachate concentrations) to determine if leachates of *S*. *fluitans* and *S*. *natans* modify larval swimming behavior under laboratory conditions. A ten-minute video was used to obtain global (larval displacements and inactivity around the tank) and specific (larval movements at the surface and at the bottom of the tank) trajectometry indexes (e.g., distance, time, and speed of larvae per treatment) and the frequency of 25 types of behavioral displacements. Posteriorly, larval activity was observed for an additional 20 min (hereafter called “post-observations”). Larvae with leachates swam slower, displaced over shorter distances, and were positively geotactic. In the videos, the majority of behavioral displacements in larvae exposed to leachates displayed low frequencies, while during post-observations most larvae (80–100%) were motionless. Our results revealed that larval dispersal capacity may be reduced and even stopped (i.e., motionless) in the presence of *Sargassum* leachates. Additionally, a high percentage of *A*. *palmata* larvae exposed to *Sargassum* leachates swam in a spiral pattern, which could be a symptomatic behavior of coral larvae exposed to pollution. This is the first study that demonstrates the negative effects of *Sargassum* leachates on the swimming behavior of coral larvae. Given that massive arrivals of *Sargassum* appear to be a new frequent disturbance in the Caribbean Sea, further study of how coral early life stages (including larvae) respond to lower concentrations of leachates are necessary to reveal what occurs in natural settings. Under this possible scenario, we showed that *Sargassum* arrivals in the Caribbean might be a major threat for sexual coral reproduction.

## Results

### Global and specific trajectometry indexes

In total, 16 trajectometry indexes were evaluated in *A*. *palmata* larvae: 10 global and 6 specific. Irrespective of concentration, *Sargassum* leachates had negative effects on 8 global and 4 specific trajectometry indexes. *Sargassum* leachates affected larval performance: larvae in contact with leachates moved slower (hypoactive) and swam shorter distances, decreasing dispersion capacity. Additionally, larvae in contact with leachates became positively geotactic, while larvae in control and stain treatments became negatively geotactic.

#### Global trajectometry indexes

**Figure 2:**
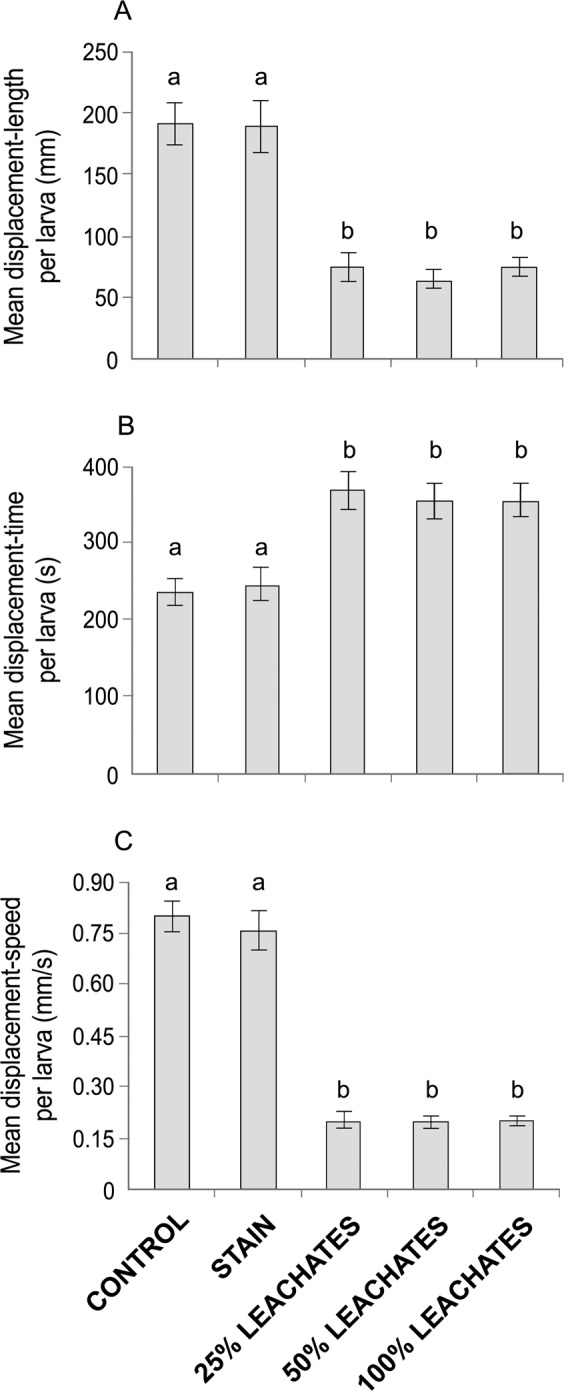
Global indexes evaluated through five treatments (control, stain, and 25%, 50% and 100% leachates) in *Acropora palmata* larvae: (**A**) Mean displacement-length, (**B**) mean displacement-time, and (**C**) mean displacement-speed per larva. Letters above the bars indicate significant differences according to *post hoc* analyses. Error bars represent standard error. See text for more details.

The displacement-length of larvae in contact with leachates (25%: 75.3 ± 11.0 mm, 50%: 65.3 ± 7.1 mm, and 100%: 74.9 ± 8.3 mm) was 2.5 times lower than larvae in both the control (190.4 ± 16.8 mm) and stain (188.6 ± 20.9 mm) treatments (KW: H_(4,244)_ = 59.04, *P* < 0.001; Fig. 2A). In contrast, the displacement-time of larvae exposed to leachates (25%: 369.0 ± 23.6 s, 50%: 355.7 ± 22.8 s, and 100%: 355.1 ± 21.0 s) was greater than larvae without leachates (control: 235.1± 16.4 s, and stain: 246.5 ± 20.3 s; ANOVA: F_(4,244)_ = 9.80, *P* < 0.001; Fig. 2B). Larvae exposed to leachates were three or more times slower with regard to their displacement-speed (25%: 0.21 ± 0.02 mm/s, 50%: 0.19 ± 0.02 mm/s, and 100%: 0.21 ± 0.02 mm/s) compared with larvae in the absence of leachates (control: 0.80 ± 0.05 mm/s, and stain: 0.75 ± 0.06 mm/s; H_(4,244)_ = 144.24, *P* < 0.001; Fig. 2C).

**Figure 3:**
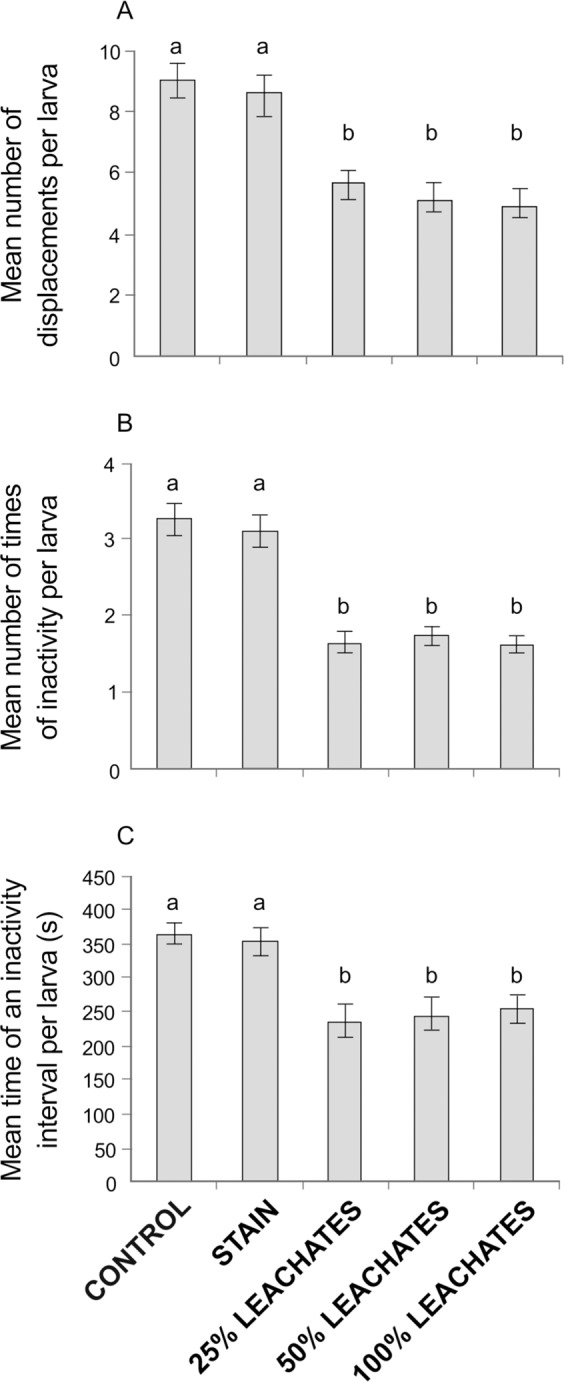
Global behavior indexes of coral larvae evaluated in five treatments (control, stain, and 25%, 50% and 100% leachates): (**A**) Mean number of displacements, (**B**) mean number of times of inactivity, and (**C**) mean time of an inactivity interval per larva. Letters above the bars indicate significant differences according to *post hoc* analyses. Error bars represent standard error.

The number of displacements (25% = 5.6 ± 0.5, 50% = 5.1 ± 0.5, and 100% = 4.9 ± 0.5 vs. control = 9.0 ± 0.6, and stain = 8.5 ± 0.7; H_(4,244)_ = 37.70, *P* < 0.001; Fig. 3A) and the number of times of inactivity (25% = 1.7 ± 0.1, 50% = 1.7 ± 0.1, and 100% = 1.6 ± 0.1 vs. control = 3.3 ± 0.2, and stain = 3.1 ± 0.2; H_(4,244)_ = 68.88, *P* < 0.001; Fig. 3B) of larvae exposed to leachates were almost half that of with larvae in the absence of leachates. The time of an inactivity interval per larva exposed to leachates (25%: 236.8 ± 23.8 s, 50%: 246.2 ± 23.2 s, and 100%: 255.1 ± 20.5 s) was at least 100 s slower than larva without leachates (control: 364.9 ± 16.4 s, and stain: 353.5 ± 20.3 s; H_(4,244)_ = 32.34, *P* < 0.001; Fig. 3C).

**Figure 4:**
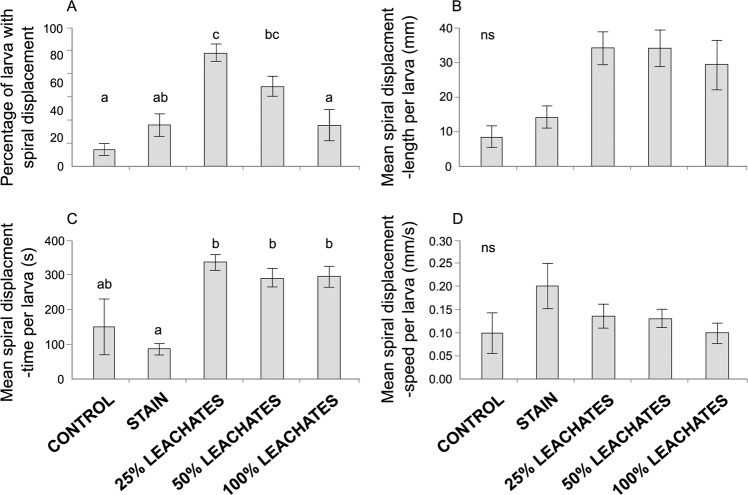
The four trajectometry indexes used to evaluate spiral movement in *Acropora palmata* larvae exposed to five treatments (control, stain, and 25%, 50% and 100% leachates). (**A**) Percentage of larvae with spiral displacement. (**B**) Mean spiral displacement-length per larva. (**C**) Mean spiral displacement-time per larva. (**D**) Mean spiral displacement-speed per larva. Letters above the bars indicate significant differences according to *post hoc* analyses; ns = not significant. Error bars represent standard error.

Spiral movement is characterized as a rotation movement on a larva’s own axis either clockwise or counterclockwise; however, this movement was observed with little or without displacement in the tank. Spiral movement responded differentially in two of the four trajectometry indexes evaluated among treatments (Fig. 4). The percentage of larvae with spiral displacement in 25% (82 ± 6%) was double or more than in the control (12 ± 4%), stain (30 ± 8%), and 100% exposure (30 ± 11%) treatments, but 50% leachates (58 ± 7%) was similar to the stain and 25% leachates (H_(4,107)_ = 25.76, *P* < 0.001; Fig. 4A) treatments. The spiral displacement-time per larva in leachate treatments (25%: 336.4 ± 22.7 s, 50%: 291.9 ± 27.8 s, and 100%: 296.0 ± 30.9 s) was three times greater than in the stain treatment (86.7 ± 14.9 s), while the control (152.0 ± 80.4 s) was similar to the stain and leachate treatments (H_(4,107)_ = 32.06, *P* < 0.001; Fig. 4C). In contrast, the spiral displacement-length (8.4–34.3 ± 3.1–7.3 mm; H_(4,107)_ = 12.29, *P* > 0.05; Fig. 4B) and the spiral displacement-speed (0.10–0.20 ± 0.02–0.05 mm/s; H_(4,107)_ = 5.46, *P* > 0.05; Fig. 4D) per larvae did not different among treatments.

#### Specific trajectometry indexes

**Figure 5:**
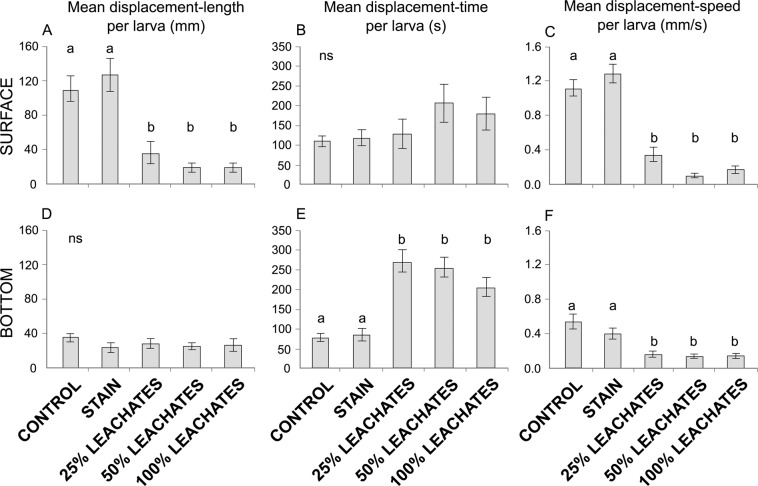
Specific trajectometry indexes per larva of *Acropora palmata* swimming at the surface (**A–C**) and at the bottom (**D**–**F**) of the tank exposed to five treatments (control, stain, and 25%, 50% and 100% leachates) (**A**,**D**) Mean displacement-length per larva. (**B**,**E**) Mean displacement-time per larva. (**C**,**F**) Mean displacement-speed per larva. Letters above the bars indicate significant differences according to *post hoc* analyses; ns = not significant. Error bars represent standard error.

Displacement-length of larvae at the surface in leachate exposure treatments (25%: 36.5 ± 13.1 mm, 50%: 18.6 ± 5.1 mm, and 100%: 19.4 ± 4.9 mm) was at least three times shorter than larvae without leachates (control: 111.1 ± 15.0 mm, and stain: 127.7 ± 19.5 mm; H_(4,89)_ = 36.77, *P* < 0.001; Fig. 5A). Displacement-time per larva did not differ among treatments (110.0–206.2 ± 13.7–48.1 s; H_(4,89)_ = 5.07, *P* > 0.05; Fig. 5B). Displacement-speed per larva exposed to leachates (25%: 0.34 ± 0.08 mm/s, 50%: 0.10 ± 0.02 mm/s, and 100%: 0.17 ± 0.04 mm/s) was at least three times slower than larvae without leachates (control: 1.12 ± 0.10 mm/s, and stain: 1.29 ± 0.11 mm/s; F_(4,89)_ = 57.51, *P* < 0.001; Fig. 5C).

Displacement-length per larva at the bottom of the tank did not differ among treatments (24.5–35.8 ± 5.0–7.3 mm; H_(4,168)_ = 3.73, *P* > 0.05; Fig. 5D). Displacement-time per larva exposed to leachates (25%: 276.5 ± 27.9 s, 50%: 260.8 ± 25.1 s, and 100%: 208.4 ± 24.3 s) was three times higher than larva in both the control (78.9 ± 10.3 s) and stain (86.0 ± 15.4 s; H_(4,168)_ = 57.69, *P* < 0.001; Fig. 5E) treatments. Conversely, displacement-speed per larva exposed to leachates (25%: 0.16 ± 0.03 mm/s, 50%: 0.14 ± 0.02 mm/s, and 100%: 0.14 ± 0.03 mm/s) was at least two times slower than larvae in both the control (0.54 ± 0.09 mm/s) and stain (0.40 ± 0.06 mm/s; F_(4,168)_ = 8.17, *P* < 0.001; Fig. 5F) treatments.

### Frequency of behavioral displacements

**Figure 6:**
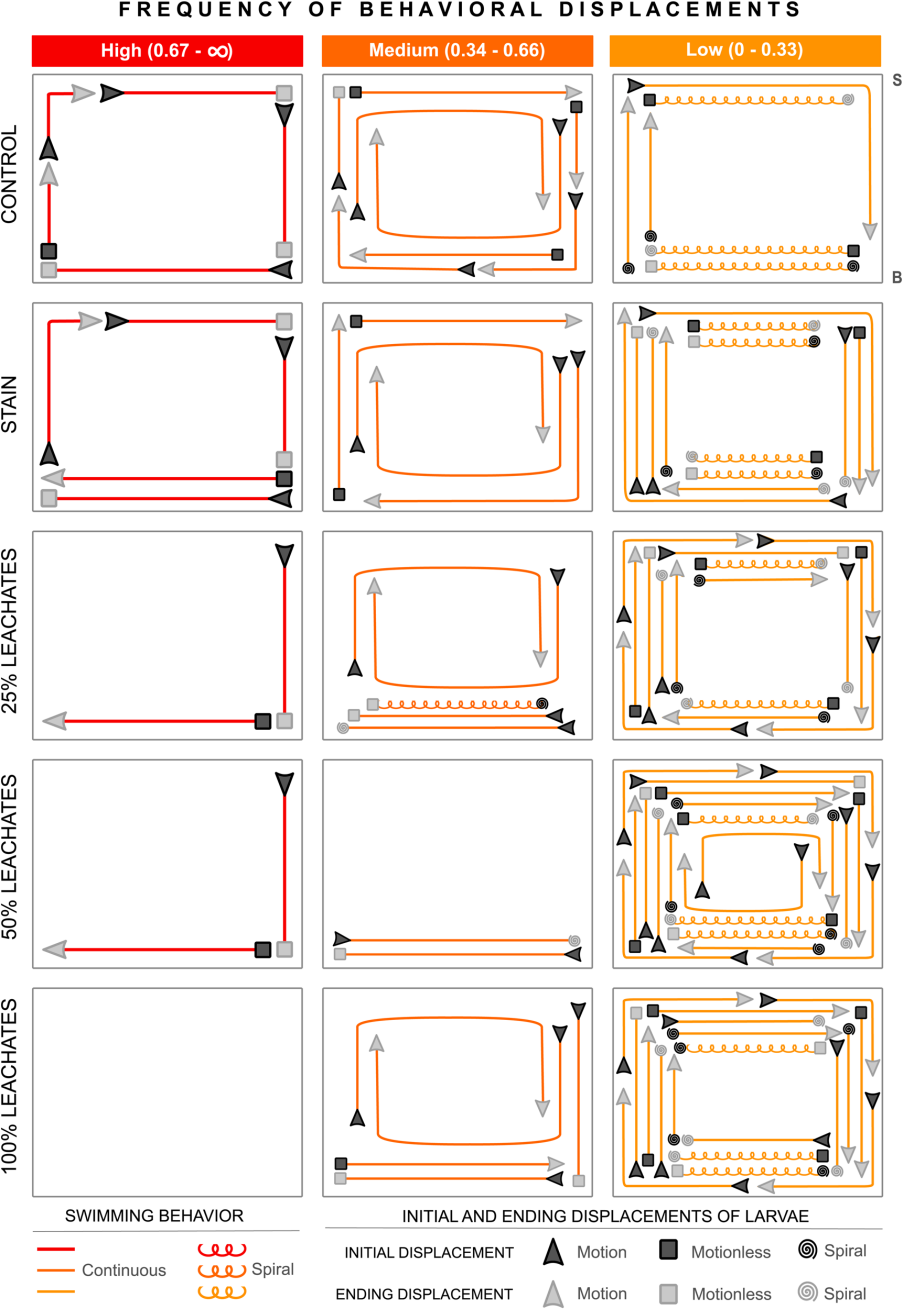
Schematic representation in the tank of the frequency of behavioral displacements of *Acropora palmata* larvae exposed to five treatments (control, stain, and 25%, 50% and 100% leachates). The frequency of each type of displacement per treatment was assigned to one of three ranks: high (0.67–∞), medium (0.34–0.66), and low (0.00–0.33). S = seawater surface; B = bottom of the tank. See Material and Methods for more details about the description of the beginning and the end of each displacement.

Overall, *A*. *palmata* larvae displayed 25 different types of behavioral displacements, but not all of them occurred in all treatments. The frequency with which each type of behavioral displacement occurred per treatment was assigned to one of three ranks: low (0.00–0.33), medium (0.34–0.66) and high (0.67–∞) (Fig. 6 and Suppl. Table 1). Larvae either swam continuously or in spiral movements, but there were also moments of inactivity (called ‘motionless’). Larvae started and ended displacements in a spiral motion, any motion other than a spiral, or remained motionless (Fig. 6 and Suppl. Table 1). 18 types of larval behavioral displacements were observed in the control treatment (G = 0.9, df = 2, *P* > 0.05) at different levels of frequency (5 high, 8 medium, and 5 low); 22 types (5 high, 5 medium, and 12 low) in the stain treatment (G = 4.1, df = 2, *P* > 0.05); 22 types (2 high, 4 medium, and 16 low) in the 25% leachates treatment (G = 14.9, df = 2, *P* < 0.001); 24 types (2 high, 2 medium, and 20 low) in the 50% leachates treatment (G = 25.5, df = 2, *P* < 0.001); and 23 types (5 medium, and 18 low) in the 100% leachates treatment (G = 7.7, df = 1, *P* < 0.001). These results indicate that most of the different types of behavioral displacements observed in larvae exposed to leachates occurred less frequently (belonged to the low rank), while five types of behavioral displacements in the control and the stain treatments occurred quite frequently (belonged to the high rank). Spiral movement was related to 11 of the 25 types of behavioral displacements; most of them belonged to the low frequency rank. Four different spiral behavioral displacements occurred in the control treatment, eight in the stain treatment, nine in the 25% leachates treatment, nine in the 50% leachates treatment, and 10 in the 100% leachates treatment (Fig. 6 and Suppl. Table 1).

### Post-observations

During post-observations, all larvae in both the control and the stain treatments were active swimmers, mainly exploring the surface at a high velocity. In contrast, larvae exposed to leachates moved slowly and eventually became motionless, depending on the leachate concentration. Most larvae in contact with leachates became motionless at the end of 20 min: 80% of larvae in the 25% treatment, and 100% of larvae in 50% and 100% leachates.

#### Control

Throughout the 20 min of observation, all of the larvae were active (swimming quickly) compared to the larvae in the presence of leachates. Larvae mainly swam on the surface and bottom, but they also had the capacity to swim upward, downward, and side to side.

#### Stain

The activity of the larvae in contact with the food colorant was similar to larvae in the control. During the 20 min of observation, all larvae moved rapidly, primarily at the surface and at the bottom; however, they also moved up, down, and side to side.

#### 25% leachates

During the first 5 min, almost 80% of the larvae were active (moving up and down or from side to side), but they were slower in comparison to the control and stain treatments. Within 5 to 10 min, larval activity decreased (i.e., hypoactive) or stopped (i.e., motionless), and larvae floated (on the surface or in the middle of the water column) or sunk to the bottom. Within 15 min, 80% of the larvae were motionless, mostly on the bottom, but also at the surface or in the middle of water column. In the last 5 min (i.e., 20 min), larvae were again on the bottom, in the middle of the water column or at the surface. 80% of the larvae were motionless and 20% of the larvae displayed extremely low mobility.

#### 50% leachates

During the first 10 min, mobility was low in 60% of the larvae. They were located either close to the bottom or in the middle of water column. During the last 10 min, all larvae were motionless, lying on the bottom or suspended in the middle of the water column, but not at the surface.

#### 100% leachates

During the first minute, all larvae reduced their activity and at the end of 5 min, 70% of larvae were motionless and 30% hypoactive. These larvae moved up and down or erratically. After 5 min, all larvae lost mobility (i.e., completely stopped), and were located at the surface or on the bottom.

## Discussion

This is the first study that shows how pelagic *Sargassum* leachates affect coral larval behavior, specifically, the swimming performance of *A*. *palmata* larvae. Most of the trajectometry indexes (both global and specific) showed differences among larvae exposed to leachates (25%, 50% and 100%) vs. without leachates (control and stain). Global trajectometry indexes highlighted general aspects of larval behavior, which included moments of activity and inactivity in any direction. In contrast, specific trajectometry indexes revealed detailed information about continuous displacements of larvae at the surface and on the bottom. Coral larvae are poor swimmers and other factors (e.g., current direction and wind) often play more of a role in larval dispersion^18^. Nonetheless, if only larval movements are considered, vertical and horizontal displacements may reveal which one plays a more important role. In this context, leachates limited the swimming abilities of *A*. *palmata* larvae since larvae swam shorter distances at the surface, spent more time on the bottom, and moved at slower speeds. The reduction of displacement-speed per larva (swimming speed decreased up to four times) exemplified how exposure to leachates negatively affects larvae. Stressful environments may influence the performance of coral offspring^39^, since larvae respond to environmental changes^17^. In this study, hypoactive larvae in leachates became positively geotactic (i.e., spent more time at the bottom instead of at the surface). This also occurs in coral larvae living in stressful conditions^17^. Larvae of *A*. *palmata* can be competent up to 3 weeks (Szmant unpubl. data, see^40^), but the slower speed observed in videos and the loss of larval activity in the presence of leachates during post-observations suggest that larval dispersal capacity is highly reduced. Coral larval dispersion normally occurs in the water column (including the surface)^18^ during the first few days^17^, but as time goes on, larval speed^41^ and energy reserves decrease because larvae lack zooxanthellae^42^. Conversely in our study, in the first few days, competent larvae showed slower speed and positively geotactic behavior (due to dispersion associated with the surface^18^ observed in videos, and loss of larval activity in the presence of leachates during post-observations suggest that larval dispersal capacity of *A*. *palmata* is highly reduced. The massive arrival and decomposition of pelagic *Sargassum* may create unfavorable conditions^17^ that make it impossible for *A*. *palmata* larvae to escape. Stressful environmental conditions and benthic biota may harm early life stages of many organisms. The presence of oil, oil dispersants, nutrient enrichment, increase of ocean acidification, ultraviolet rays and temperature, and the presence of allopathic algae compounds, macroalgae and cyanobacteria might reduce settlement and survivorship of coral larvae^19,22–25,37,43,44^. *Sargassum* leachates are a new disturbance that affects the behavior of *A*. *palmata* larvae. Therefore, the replacement of *A*. *palmata* colonies from sexual reproduction might be further reduced.

Little is known about spiral movements like those observed in coral larvae exposed to *Sargassum* leachates. These particular movements have been reported in larvae of marine invertebrates such as sponges^45,46^, mussels^47^ and corals^20,48,49^. Spiral movement is usually described as a clockwise rotation in one place or with a slow or erratic displacement^49^. Under increased pressure, coral larvae may swim upwards in spiral, as a compensatory response^48^. Spiral motion has also been identified as a negative response to different concentrations of oil dispersants. Oil produced disoriented larval spiral movements after several hours^20^. In our study, larvae that moved in a spiral motion were present in all treatments. The percentage of larvae with spiral displacements was the metric that best exemplified the negative effects of *Sargassum* leachates. The percentage of larvae displaying this symptomatic behavior was high under 25% and 50% concentrations (82% and 58%, respectively). However, the low percentage of spiral swimming larvae in the control and the stain treatments could be considered to be normal (12% and 30%, respectively) because, even under non-stressful conditions like temperature (28 °C), a small percentage of *A*. *palmata* embryos/larvae (11–22%) have abnormalities^50^. The low percentage of spiral swimming larvae exposed to 100% leachates (30%) was unexpected. Chemical compounds released by pelagic *Sargassum* (not measured in this study) in the 100% leachates treatment could also modify larval behavior. For instance, waterborne macroalgae has the ability to modify larval behavior, both decreasing and delaying the recruitment of the coral *Acropora millepora*^51^. Additionally, larvae exposed to 100% leachates were the only ones that did not display a high frequency of any of the 23 behavioral displacements observed. Larval speed when swimming in a spiral is constant regardless of treatment (0.10–0.20 mm/s). This speed is similar to the speed of larvae swimming in leachates (0.19–0.21 mm/s), which suggests that swimming in a spiral motion is a negative behavior that reduces larval dispersion. It seems to have a greater influence on larvae that continued to rotate on their own axes, without the onward motion produced in seawater (i.e., with stationary movements). Marine invertebrates may also display spiral movements as a result of light exposure. Less active sponge larvae swam in a spiral or did not respond at all when faced with a stimulus such as light and active larvae responded negatively to light^45^. In our study, turbidity of leachates did not play an important role because larvae in the stain (similar color of 100% leachate) treatment showed opposite responses in most of the metrics evaluated. This highlights that some physical and chemical parameters of leachates may negatively affect *A*. *palmata* larvae. Thus, *Sargassum* leachates affect swimming performance and possibly alter sensory perception similar to when fish are exposed to metals^52^.

During post-observations, larval activity under the influence of leachates decreased as leachate concentration increased. After 10 min of video recordings, 20 min of post-observations of leachate exposure was enough to cause all coral larvae to become motionless in 50% (after10 min) and 100% leachates (after 5 min), while 80% of the larvae were motionless in 25% leachates (after 15 min). Nevertheless, if larvae were exposed over a longer time period to 25% leachates, they would probably have become motionless as well. We cannot be certain that motionless larvae at the bottom settled or died; but slower speeds, lower dispersion, and low frequency in most of the behavioral displacements might indicate that leachates eventually may kill *A*. *palmata* larvae. Coral larvae exposed to dispersants may survive more than 96 h, but those larvae (with abnormalities, and atypical swimming and searching behavior of substratum) sooner or later died^20^. Therefore, larvae exposed to leachates may reduce dispersion, decreasing their capacity to find better conditions to settle and recruit.

In this study, values of DO (1.51 mg L^−1^) and pH (7.5) in the 25% leachate concentration were similar to those reported in two Mexican Caribbean reefs^10,11^ at similar distances to where *A*. *palmata* gametes were collected (from 60 m away from the coast; Fig. 1 and Suppl. Fig. 1). On August 2015, a southern coral reef registered values of ~1.00 mg L^−1^ of DO 50 to 100 m away from the coastline, while a pH of 7.4 was recorded in the highest impact zone (with *Sargassum* in decomposition) up to ~50–70 m^11^. On May 2018, values of 1.90 mg L-1 of DO were recorded at a northern coral reef (closer to our study site), 130 m away from the coastline^10^. Because larvae responded negatively when in contact with leachates regardless of their concentration, *A*. *palmata* larvae exposed to leachates in natural conditions may also react negatively. However, it is possible that 25% leachates (i.e., the lowest concentration in our study) exceeded the threshold that larvae can withstand. Thus, we suggest that future assays should evaluate lower leachate concentrations and over longer periods of exposure to obtain results that are closer to those that occur in the field, and to determine at what *Sargassum* leachate concentration corals are affected. The most *Sargassum* arrived between May to July on 2018 in the Caribbean Sea^9^, but high fauna mortality associated with its arrival was observed from May to September^11^. Because our study was carried out on August 2018, we believe that *Sargassum* leachates and the decomposition of fauna may exert negative effects on the spawning of *A*. *palmata* due to the decrease in seawater quality.

Leachates derived from pelagic *Sargassum* decomposition is an additional source of stress for *A*. *palmata* larvae. However, leachates could affect other early life stages such as: fecundity, embryogenesis, settlement, and recruitment. Under laboratory conditions, our results reveal the potential effects of *Sargassum* leachates. It is possible that similar effects occur in the field because the highest arrival of *Sargassum*^9,10^ with high fauna mortality^10^ (a factor that is not evaluated in this study) partially coincided with the reproductive season of several broadcasting coral species in the Caribbean (July-October)^27^ like *A*. *palmata* (July-September)^27,28^. *Sargassum* leachates may also reduce the input of sexual recruits of *A*. *palmata* and other coral species that live in shallow environments. Another concern for the early life stages of corals is the possibility that drifting *Sargassum* could trap gametes and embryos because of their buoyancy^38^, and larvae due to their negative geotactic behavior^17,18^ in the first hours^53^, which may increase mortality. If massive arrival of *Sargassum* on the coast of the Mexican Caribbean results in the death of adult stony corals (both brooders and broadcasting species)^11^, drifting *Sargassum* and leachates may have more negative effects on corals due to higher mortality rates in the early life stages^15,54^. Since some key reef-building (long-living large colonies that offer a high structural complexity like *A*. *palmata* in this study) and opportunistic stony coral species (short-lived medium or small colonies that do not contribute with the same structural complexity in coral reefs, like *Porites astreoides*)^55,56^ have been affected by *Sargassum* arrivals^11^, genetic diversity and the extension of coral cover might not increase. Another concern is the implementation of barriers that are used to trap *Sargassum* in reef lagoons (placed in the northern Mexican Caribbean^11^). These barriers may increase leachate concentration if *Sargassum* collection does not occur before decomposition begins (within hours or days). Our results identify potential effects that *Sargassum* leachates could have on *A*. *palmata* larvae in the field and open opportunities to research problems caused by the massive arrivals of pelagic *Sargassum* spp. in the Caribbean region. The resilience of corals (both reef-building and opportunistic species) may be tested if *Sargassum* arrivals, which have already occurred since 2011 in the Caribbean^9^, continue.

## Materials and Methods

### Study area

Gamete bundles of *Acropora palmata* were collected at Punta Venado (20°31′57″N, 87°10′26″W), the northern part of Quintana Roo, in the Mexican Caribbean (Suppl. Fig. 2). Punta Venado is part of the Mesoamerican Barrier Reef^57^, which is considered to be the second largest coral barrier after the GBR in Australia. *A*. *palmata*, an abundant reef-building coral species in the northern part of the Mexican Caribbean^23^, may experience contact with *Sargassum* leachates (Suppl. Fig. 3). *A*. *palmata* colonies are found 60 m away from the coastline at Punta Venado (Fig. 1).

### Elaboration of coral larvae tanks and substrates

Small handmade tanks (5 × 5 × 1.2 cm) for coral larvae were constructed with slides of 7.5 × 5 cm, which were cut and glued (with Norland Optical Adhesive 81) in the optical laboratory at Centro de Investigación Científica y de Educación Superior de Ensenada, Baja California (CICESE). To prepare substrates, polystyrene rectangles (5 × 1.2 cm) were cut and sanded, while tiles of limestone were mashed and filtered in a strainer to obtain small grains. Later, limestone grains were glued on polystyrene with non-toxic silicone (Sista F109). Substrates were submerged in a tank with the presence of coralline crustose algae three weeks prior to coral spawning to favor recruitment (called “conditioned substrates”).

### Sample collection

Coral egg-sperm bundles from ten *A*. *palmata* colonies were collected at 22:06 h on August 3, 2018 (six days after the full moon). Due to the buoyancy of gametes, egg-sperm bundles were collected with inverted conical nets (made with 100 μm filter mesh), adapted from a transparent flask to contain gametes. Flasks were capped and transported (upside down) to the boat without shaking in order to avoid breaking packages. Once all the containers were gathered, the gamete bundles and seawater were gently released and mixed in a transparent container with 10 L of filtered seawater to begin fertilization. Gametes were transported to the Xcaret Aquarium to stop fertilization (~2 h) and were washed several times with seawater (filtered with numerous meshes (from 100 to 5 µm) and UV filters) to eliminate excess sperm, avoiding polyspermia.

### Coral embryos and larvae culture

After fertilization, 3 ml of embryos were transferred to three incubators (1 ml per incubator). Each incubator consisted of a 20 L plastic bucket divided in half, separated with a micro nylon filter mesh (75 μm) that allowed for water to interchange, but not the exit of embryos. The incubators were semi-submerged in a fiberglass tank (1 × 1 × 1 m) almost full of seawater with closed flow. An in-line pump enabled seawater recirculation through sprayer nozzles inside incubators to create smooth irrigation and avoid embryo agglomeration.

### Leachate preparation

To prepare leachates, seawater was filtered through micro (from 100 to 5 µm) and UV filters and placed in another fiberglass tank (the same size as the tank used for embryos and larvae culture). In the tank, filtered seawater occupied almost one quarter of its capacity and remained stagnant to use in the preparation of leachates and in the experimental treatments. Non-decomposed *S*. *fluitans* and *S*. *natans* were collected and washed several times with filtered seawater to remove sediments and organisms. Subsequently, 1.5 kg of these brown algae (wet weight) and 10 L of stagnant seawater from the tank were deposited in a smaller fiberglass tank (0.4 × 0.4 × 1 m) to obtain leachates. The three fiberglass tanks (one with embryos and larvae culture, one with stagnant filtered seawater, and one with a mixture of algae and seawater to obtain leachates) were left outdoors, side by side, in a space without walls, but with a translucent roof of polycarbonate (~140 m^2^). After the *Sargassum* had soaked in the seawater for 3.5 days, leachates were obtained in order to prepare different concentrations (25%, 50% and 100%). Later, leachates were filtered with a nylon mesh and any particles larger than 75 μm were removed to better visualize coral larvae in the experiment.

### Experimental design

**Figure 7:**
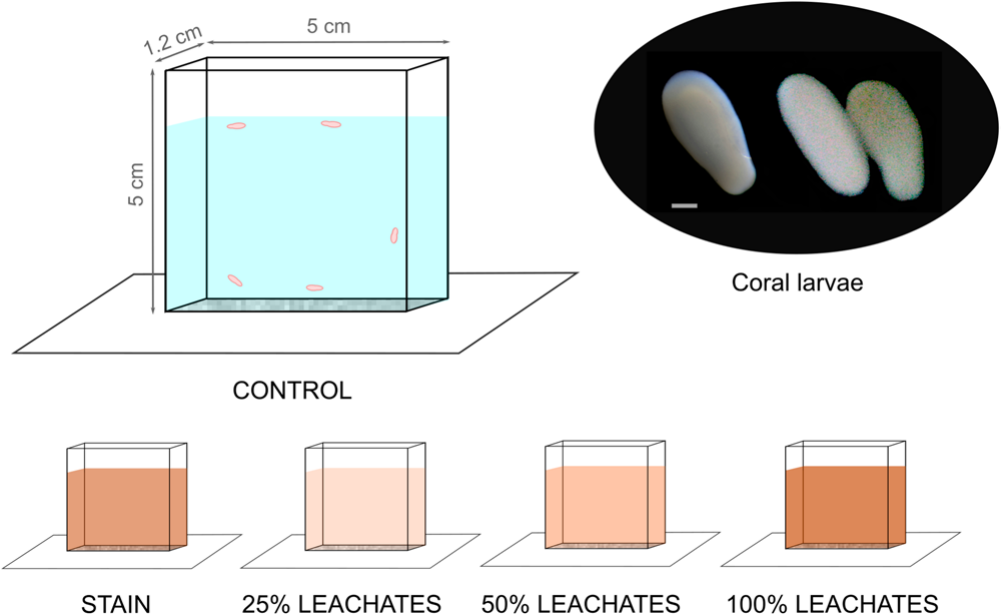
Experimental design to evaluate larval swimming behavior through five treatments (control, stain, and 25%, 50% and 100% leachate concentrations). Scale: 200 µm. See text for more details. Larvae were taken by H. Bahena-Basave (left larva) and by N. P. Cetz-Navarro (middle and right larvae).

The effect of *Sargassum* leachates on the larval swimming behavior of *A*. *palmata* was evaluated through the use of five treatments on August 08 2018 during daylight hours (11:30–14:00 h) since larvae are more active during this period (pers. obs.). Treatment 1 (hereafter called “control”) consisted in larvae exposed to filtered seawater. Treatment 2 (hereafter called “stain”) consisted in larvae exposed to filtered seawater with caramel artificial food coloring to simulate the color of the highest concentration of leachate (100%) to evaluate if color affects coral larvae. In the last three treatments (T3-T5), larvae were exposed to different leachate concentrations to evaluate their impact on *A*. *palmata*: T3 (hereafter called “25% leachates”) was comprised of larvae exposed to a 25% concentration of leachates, T4 (hereafter called “50% leachates”) contained larvae exposed to a 50% concentration of leachates, and T5 (hereafter called “100% leachates”) included larvae exposed to a100% concentration of leachates. Five competent coral larvae, of similar size and shape (i.e., bowling pine or elongated) were deposited on the fifth day after fertilization per tank or replicate. Each small tank contained a conditioned substratum of limestone at the bottom and 25 ml of filtered seawater, with or without leachates, depending on the treatment (Fig. 7). In total, 250 larvae were used (5 larvae × 5 treatment × 10 replicates). Previously, seawater of each treatment was placed in a plastic bottle; in total, five bottles (one per treatment) were semi-submerged in a tank (3 × 2 × 2 m) with continuous seawater flow in order to ensure that the seawater within the bottles remained at a constant temperature among treatments. Before recording each video, practical salinity unit (PSU), pH, dissolved oxygen (DO) and temperature (TT) were recorded using a PRO DSS probe 4-port DIGITAL. Mean data of physical parameters were obtained in the seawater used in each treatment as follows: 1) control (PSU = 38.59, pH = 8.14, DO = 6.09 mg/L, and TT = 29.40 °C); 2) stain (PSU = 38.52, pH = 8.26, DO = 6.17 mg/L, and TT = 29.40 °C); and 25% (PSU = 38.64, pH = 7.53, DO = 1.521 mg/L, and TT = 29.43 °C), 50% (PSU = 40.20, pH = 7.40, DO = 0.195 mg/L, and TT = 29.45 °C) and 100% (PSU = 41.92, pH = 7.24, DO = 0.098 mg/L, and TT = 29.45 °C) leachates. Subsequently, a 10 min video was recorded per replicate using CANON PowerShot cameras (G10, G11 and G12 models).

### Global and specific trajectometry indexes

Videos were recorded in.AVI format and projected on a 13-inch computer monitor. An acetate paper was placed on the monitor to trace (with permanent marker) all displacements and inactivity of each larva (hereafter called “trajectometry indexes”) (Table 1). Trajectometry indexes were assigned to two categories: global (10) and specific (6). Global trajectometry indexes evaluated the swimming behavior and inactivity of each larva as it moved in any direction in the tank. Specific trajectometry indexes evaluated larval swimming behavior at the surface (3) and at the bottom (3) of the tank. Larval speed was evaluated in both global and specific trajectometry indexes and was obtained from the displacement-length and -time of each larva recorded.

**Table 1:** Trajectometry indexes and their units evaluated in displacements and inactivity of *Acropora palmata* larvae exposed to five treatments (control, stain, and 25%, 50% and 100% leachates).

Trajectometry indexes	Unit
***Global***	
1) Mean displacement-length per larva	mm
2) Mean displacement-time per larva	s
3) Mean displacement-speed per larva^a^	mm/s
4) Mean number of displacements per larva^b^	count
5) Mean number of times of inactivity per larva^c^	count
6) Mean time of an inactivity interval per larva	s
7) Percentage of larvae with spiral displacement	%
8) Mean spiral displacement-length per larva	mm
9) Mean spiral displacement-time per larva	s
10) Mean spiral displacement-speed per larva	mm/s
***Specific***	
***Surface***	
11) Mean displacement-length per larva	mm
12) Mean displacement-time per larva	s
13) Mean displacement-speed per larva^a^	mm/s
***Bottom***	
14) Mean displacement-length per larva	mm
15) Mean displacement-time per larva	s
16) Mean displacement-speed per larva^a^	mm/s

^a^In this trajectometry index, only continuous activity per larva was considered. In other words, the time of inactivity (i.e., at rest or motionless) of each larva was excluded.

^b^The trajectometry per larva during each video was divided in displacements. Each displacement included a beginning and an end. When a larva modified its behavior, the last movement was defined as the end of the displacement, and the next new movement was defined as the beginning of a new displacement and so on.

^c^Number of times in which a larva was inactive or motionless during its trajectory.

### Frequency of behavioral displacements

The trajectory of each larva was traced to obtain all displacements, but moments of inactivity were also recorded per treatment. Each displacement had a beginning and an end as explained below. The first movement of each larva was defined as the beginning of a displacement; this displacement ended when the larva modified its behavior. The end of the previous displacement became the beginning of the next and ended when the larva, again, modified its behavior. This process was repeated until the last behavioral change was recorded in the space of 10 min. Each displacement (in any direction) was listed and classified into one of several types of displacements, according to what the initial and final movements were: location in the tank, larval swimming direction (surface, bottom, upward and downward), and larval activity (continuous swimming or spiraled) or inactivity (i.e., motionless). The number of times of each type of displacement per treatment was also recorded. Thus, the frequency of each type of behavioral displacement per treatment was calculated as: the number of times of each type of larval displacement occurred per treatment divided by the total number of larvae used per treatment. Each frequency value obtained was assigned to one of three ranks (low, medium, and high frequency). All the different types of behavioral displacements and their frequencies were placed in a table and represented in a diagram.

### Post-observations

After recording videos, four post-observations were recorded over a period of 20 min (i.e., at minute 5, 10, 15 and 20) to register the overall activity of larvae in each tank per treatment.

### Statistical analyses

Shapiro-Wilk and Levene tests were used to check normality and homogeneity of variances of trajectometry indexes data. For normal and homogeneous data, one-way analyses of variance (ANOVA; factor: treatment) were performed followed by Tukey *post hoc* tests. For data that lacked normality and homogeneity of variances, Kruskal-Wallis (KW) tests were performed followed by *a posteriori* test in IBM SPSS Statistics for Windows version 25 (IBM, Armonk, N.Y.). Additionally, G tests were used to compare the three ranks of all frequencies of behavioral displacements within each treatment.

## Supplementary information

Supplemental information.

Supplementary Video 

Supplementary Video

Supplementary Video
