# Supplementary material for: Leachate effects of pelagic Sargassum spp. on larval swimming behavior of the coral Acropora palmata
Source: Sci Rep. 2020 Mar 3;10:3910. doi: 10.1038/s41598-020-60864-z (PMC7054338; doi:10.1038/s41598-020-60864-z)
Supplement: Supplementary file 1 — Supplemental information. [file 41598_2020_60864_MOESM1_ESM.pdf]

Supplementary Information

Leachate effects of pelagic *Sargassum* spp. on larval swimming behavior of the coral *Acropora palmata*

Francisco Antonio-Martínez[1], Yann Henaut[1], Alejandro Vega-Zepeda[1], Ana I. Cerón-Flores[2], Rodolfo Raigoza-Figueras[2], Neidy P. Cetz-Navarro[1,3]*, Julio Espinoza-Avalos[1†]

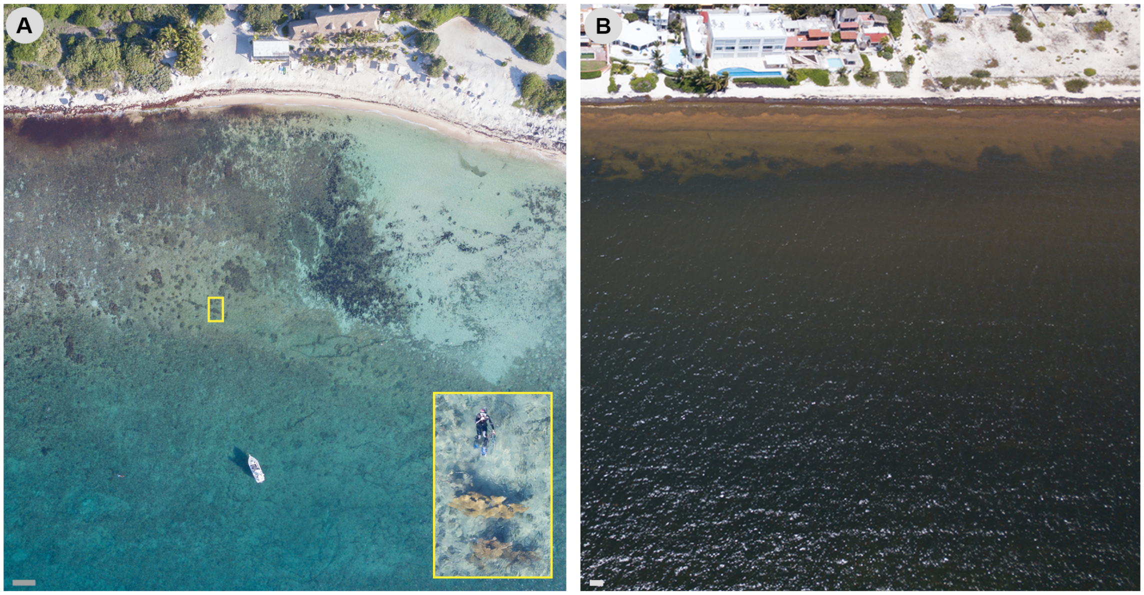

Supplementary Figure 1. Two coral reefs located in the northern Mexican Caribbean. (**A**) Study site located at Punta Venado with *Acropora palmata* colonies around 2 m wide (yellow box). (**B**) Puerto Morelos (a closer reef to our study site) with the presence of leachates, but low presence of *Sargassum* spp. at the coastline. Scale bar: 5 m. Photos by V. M. Rodríguez-Cervantes.

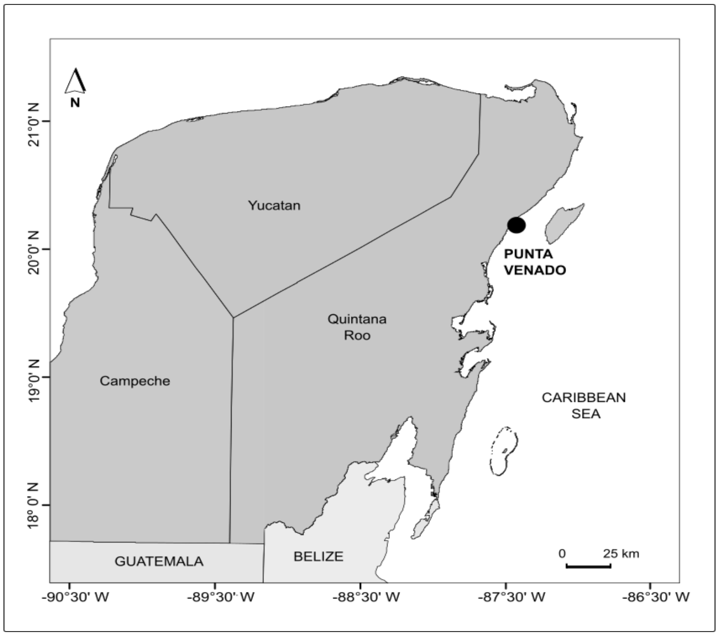

Supplementary Figure 2. Map of the study site located at Punta Venado (black dot), in the Mexican Caribbean. This map was created using ArcView 3.2 (http://www.esri.com/com/software/arcgis/arcview) and modify in Inkscape Developers, GNU, General Public License Copyright 2003-2017 (https://inkscape.org/).

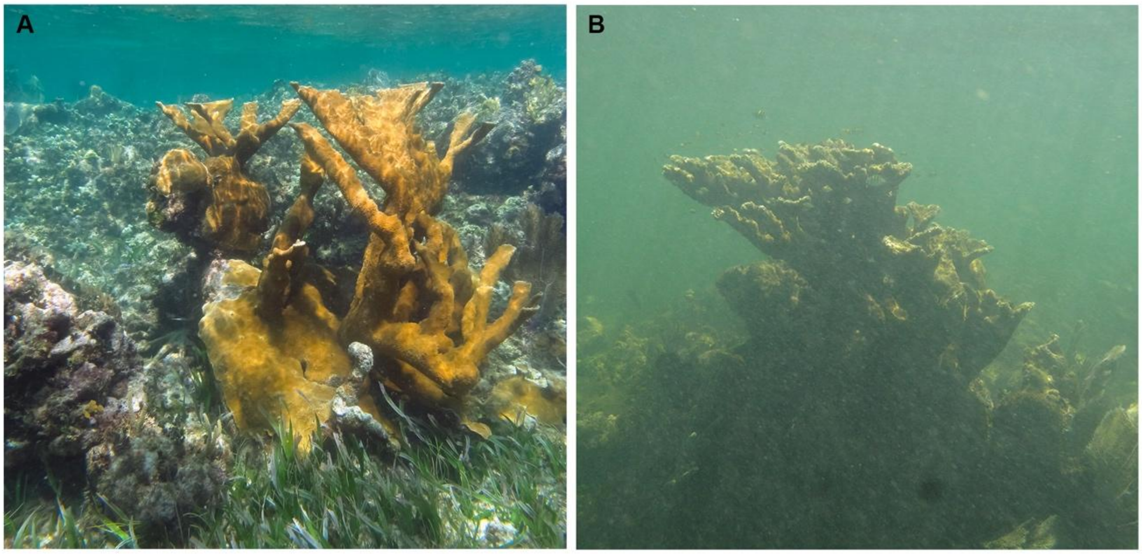

Supplementary Figure 3. Colonies of *Acropora palmata* in the Mexican Caribbean. (**A**) Colonies of *A. palmata* before the arrival of *Sargassum* spp. (**B**) Colony of *A. palmata* exposed to *Sargassum* leachates located at 1 km away from the coastline in May 2019. Photo (A) by H. Bahena-Basave, and (B) by A. Vega-Zepeda.

Behavioral displacements (beginning-end of a displacement)	Control	Stain	Leachates			KW	
			25%	50%	100%	$H_{(4,244)}$	*P*
1) Surface–motionless	1.12 ± 0.11	0.87 ± 0.09	0.04 ± 0.02	0.09 ±0.05	0.12 ± 0.05	118.37	0.001
2) Downward–motionless	0.98 ± 0.10	0.98 ± 0.08	0.76 ± 0.06	0.70 ± 0.09	0.66 ± 0.07	10.55	>0.05
3) Upward–motionless	0.96 ± 0.10	0.79 ± 0.08	0.12 ± 0.04	0.09 ± 0.04	0.24 ± 0.06	85.40	0.001
4) Bottom–motionless	0.72 ± 0.10	0.72 ± 0.10	0.42 ± 0.10	0.55 ± 0.10	0.52 ± 0.10	12.69	0.001
5) Motionless–upward	0.68 ± 0.09	0.51 ± 0.07	0.22 ± 0.05	0.13 ± 0.04	0.12 ± 0.04	45.06	0.001
6) Downward–upward	0.62 ± 0.07	0.64 ± 0.07	0.34 ± 0.06	0.21 ± 0.06	0.48 ± 0.07	24.67	0.001
7) Motionless–downward	0.62 ± 0.06	0.28 ± 0.06	0.06 ± 0.03	0.09 ± 0.04	0.14 ± 0.04	57.87	0.001
8) Motionless–bottom	0.60 ± 0.08	0.89 ± 0.05	0.78 ± 0.05	0.83 ± 0.07	0.52 ± 0.07	20.92	0.001
9) Upward–motionless	0.52 ± 0.07	0.30 ± 0.06	0.10 ± 0.04	0.17 ± 0.05	0.20 ± 0.05	25.52	0.001
10) Motionless–surface	0.46 ± 0.07	0.43 ± 0.07	NA	0.13 ± 0.04	0.16 ± 0.05	43.50	0.001
11) Upward–downward	0.40 ± 0.06	0.62 ± 0.08	0.44 ± 0.07	0.30 ± 0.06	0.42 ± 0.07	8.32	>0.05
12) Bottom–upward	0.38 ± 0.07	0.23 ± 0.06	0.14 ± 0.04	0.04 ± 0.02	0.06 ± 0.03	24.53	0.001
13) Downward–bottom	0.38 ± 0.06	0.36 ± 0.07	0.18 ± 0.05	0.11 ± 0.04	0.26 ± 0.06	12.95	0.001
14) Surface–downward	0.20 ± 0.05	0.28 ± 0.06	0.12 ± 0.04	0.11 ± 0.04	0.22 ± 0.05	6.41	>0.05
15) Motionless–spiral[S]	0.14 ± 0.04	0.02 ± 0.02	0.08 ± 0.03	0.04 ± 0.02	NA	11.10	0.001
16) Spiral–motionless[B]	0.08 ± 0.03	0.04 ± 0.29	0.34 ± 0.06	0.26 ± 0.06	0.08 ± 0.03	24.73	0.001
17) Spiral–upward	0.08 ± 0.03	0.17 ± 0.05	0.04 ± 0.02	0.11 ± 0.04	0.04 ± 0.02	7.16	0.001
18) Motionless–spiral[B]	0.06 ± 0.03	0.02 ± 0.02	0.14 ± 0.04	0.19 ± 0.05	0.04 ± 0.02	12.15	0.001
19) Spiral– bottom	NA	0.26 ± 0.06	0.26 ± 0.06	0.13 ± 0.04	NA	29.37	0.001
20) Upward–spiral	NA	0.02 ± 0.02	0.18 ± 0.05	0.09 ± 0.04	0.04 ± 0.02	16.45	0.001
21) Downward–spiral	NA	0.02 ± 0.02	0.16 ± 0.05	0.17 ± 0.05	0.08 ± 0.03	14.88	0.001
22) Spiral–motionless[S]	NA	0.02 ± 0.02	NA	NA	0.06 ± 0.03	8.38	>0.05
23) Bottom–spiral	NA	NA	0.60 ± 0.7	0.36 ± 0.7	0.12 ± 0.04	75.10	0.001
24) Spiral–surface	NA	NA	0.06 ± 0.03	0.11 ± 0.04	0.18 ± 0.05	17.63	0.001
25) Spiral–downward	NA	NA	NA	0.09 ± 0.04	0.10 ± 0.04	14.25	0.001

Supplementary Table 1. Mean values of the frequency of behavioral displacements by *Acropora palmata* larvae exposed to five treatments (control, stain, and 25%, 50% and 100% leachates). Frequency data of each type of displacement were assigned to one of three ranks: high =0.67–∞ (red underline); medium =0.34–0.66 (orange underline); and low =0.00–0.33 (yellow underline). KW=Kruskal Wallis. NA=not applicable. See text and Fig. 6 for more details. Each displacement had a beginning and an end based on movement or inactivity, swimming larval direction and location in the tank.

[S] Behavioral displacement performed on surface.

[B] Behavioral displacement performed at the bottom.
